# Acknowledgement to Reviewers of *Metabolites* in 2017

**DOI:** 10.3390/metabo8010007

**Published:** 2018-01-16

**Authors:** Metabolites Editorial Office

**Affiliations:** MDPI AG, St. Alban-Anlage 66, 4052 Basel, Switzerland

Peer review is an essential part in the publication process, ensuring that *Metabolites* maintains high quality standards for its published papers. In 2017, a total of 64 papers were published in the journal. Thanks to the cooperation of our reviewers, the median time to first decision was 18 days and the median time to publication was 47.5 days. The editors would like to express their sincere gratitude to the following reviewers for their time and dedication in 2017:
Aasly, JanLee, Hsin-ChenAldini, GiancarloLin, Randy BinAllaway, DavidLindström, MikaelAllen, DougLiu, YangAlpert, AndrewLlabrés, MercèAmato, JussaraLópez, M. G.Amos, Ruth I.J.Luchinat, ClaudioAnton, GabrieleMa, XiaochaoArita, MasanoriMangalagiu, IonelAru, ViolettaMarchetti, PhilippeBarlow, ChristopherMardinoglu, AdilBarupal, DineshMason, ShaunBaumbach, Jörg IngoMcConville, MalcolmBeccaria, MarcoMendes, Marta V.Behrends, VolkerMielcarek, MichalBoard, MaryMillet, OscarBoros, LaszloMomken, ImanBrozinski, Jenny-MariaMor, MarcoBruce, Clinton R.Murtola, TeemuBruggeman, FrankNaito, YujiBrundin, PatrikOrlandi, RosariaBueschl, ChristophPalsson, Bernhard Ø.Câmara, JoséPark, SangkyuCampos, BrunoPark, Junyoung O.Cao, MingshuParrino, VincenzoCappello, TizianaPechlivanis, AlexandrosChappell, JosephPérez, SandraCheca, AntonioPetras, DanielChekmeneva, ElenaPhillips, DavidChen, ChiPietropaoli, Anthony P.Cheng, Yuan-BinPinker, KatjaChetwynd, AndrewPiva, TerrenceChianese, GiuseppinaPorcar, ManuelChmurzynska, AgataPortais, Jean-CharlesChristians, UwePrudent, MichelCorbet, CyrilQin, ZhiqiangCox, I. JaneRachek, LyudmilaCox, JamesRaftery, DanCuleddu, NicolaReshkin, Stephan JoelCuperlovic-Culf, MiroslavaRey-Stolle, FernandaD’Archivio, Angelo AntonioRoberto, ConsonniD'Alessandro, AngeloRochat, BertrandDaskalaki, EvangeliaRogers, SimonDe La Fuente, Macarena I.Rosebrock, Adam P.Demarquoy, JeanRotili, DanteDenko, NicholasRöttger, RichardDerfuss, TobiasRovnyak, DavidDesouza, DavidRoznere, IevaDixon, Robert BrentSalim, VonnyDragsted, Lars OveSanchez, LauraDriscoll, James JSanchez Alcazar, José ADuchemin, SandrineSantucci, AnnalisaDuncan, SylviaSchefold, Joerg C.Dunphy, Brendon JohnSchena, F. P.El-Aneed, AnasSimón-Manso, YamilElena-Herrmann, BénédicteSone, HidekoEllis, Robert P.Spiess, AntjeFanizzi, Francesco P.Stefanon, BrunoFasulo, SalvatoreStokić, EditaFrizzell, NormaStringer, Kathleen A.Gatti, LauraSu, HuaboGeng, FengSullivan, LucasGesell, AndreasSurguchov, AndreiGilli, FrancescaSuzuki, TakafumiGonzález-Domínguez, RaúlSvastova, EliskaGopalan, VenkatSwiezewska, EwaGowda, G. A. NaganaTaïeb, DavidGreaves, RondaTaiti, CosimoGu, HaiweiTang, YinjieHalama, AnnaTaylor, MatthewHalbedel, SvenThakur, Sunitha BHamilton, Joshua J.Thomaidis, Nikolaos S.Han, BeomsooTiziani, StefanoHarper, JamesTolstikov, VladimirHernandez-Hierro, José MiguelTsunoda, MakotoHoene, MiriamTurano, PaolaHuang, HaoVan Velzen, Ewoud J. J.Iorio, EgidioVerpoorte, RobertJaumot, JoaquimVolmer, DietrichJayawardana, KaushalaVon Elert, EricJimenez Del Val, IoscaniWahli, WalterJohansson, Pär I.Watanabe, MikiJourdan, FabienWatson, DavidJoyce, Susan A.Weljie, AalimJunker, BjoernWendt, ChristineKato, YasumasaWest, CarolineKeijer, JaapWhiteson, KatrineKellogg, JoshWilliams, StephenKelly, Rachel S.Williamson, MikeKhetani, SalmanWilson, ThomasKim, Young-MoWong, Ka-ChunKim, JayoungXu, YunKoller, MartinYetukuri, LaxmanKonhilas, JohnYokoo, HirokiKouremenos, KonstantinosYoon, SeongkyuKriebardis, Anastasios G.Yu, FangKrššák, MartinYu, JianKudłak, BłażejZalubovskis, RaivisLalk, MichaelZanghellini, JürgenLatendresse, MarioZucker, JeremyLee, Do Yup


